# *Entamoeba gingivalis* induces gingival cell death, collagen breakdown, and host immune response via VAMP8/-3-driven exocytosis pathways

**DOI:** 10.1128/iai.00005-25

**Published:** 2025-03-21

**Authors:** Lea Rosenfeld, Nico Neumann, Xin Bao, Aysegül Adam, Arne S. Schaefer

**Affiliations:** 1 Department of Periodontology, Oral Surgery and Oral Medicine, Universitätsmedizin Berlin 14903 , Berlin, Germany; University of California Davis, Davis, California, USA

**Keywords:** periodontitis, host–pathogen interactions, vesicle secretion, oral mucosa, host defense mechanisms, vesicle-associated membrane protein, entamoebiasis

## Abstract

The protozoan *Entamoeba gingivalis* commonly colonizes anaerobic periodontal pockets, induces a severe innate immune response, invades gingival mucosa, and kills epithelial cells. *E. gingivalis* infection is associated with the common oral inflammatory disease periodontitis. DNA variants in vesicle-associated membrane proteins (VAMP) -3 and -8 genes are linked to increased periodontitis risk. These genes mediate host–pathogen interactions, including mucin exocytosis to form protective barriers and matrix metalloproteinase (MMP) secretion in intestinal amoebiasis caused by *Entamoeba histolytica*. This study aimed to investigate the roles of VAMP3/8 in gingival defense and *E. gingivalis* infection mechanisms. Clustered regularly interspaced short palindromic repeats (CRISPR)-Cas9 gene editing was used to create VAMP3/8-deficient gingival epithelial cells and fibroblasts. Functional analyses included immunofluorescence, enzyme-linked immunosorbent assay (ELISA), cytotoxicity, and collagenase assays. VAMP8 co-localized with mucins in gingival epithelial cells (gECs), and VAMP3 with MMPs in gingival fibroblasts. In gECs*, E. gingivalis* infection increased mucin (MUC1: 3.6×, MUC21: 14.4×) and interleukin secretion (IL-8, IL-1B: >6×, *P* = 0.019). *VAMP8* deficiency in gECs caused higher cell death (35% vs 4% in controls) with reduced exocytosis of mucins and interleukins. Likewise, *E. gingivalis*-induced VAMP8 translocation into lipid rafts was lost in VAMP8 knockout cells, validating the participation of VAMP8 in exocytosis. In wild-type but not VAMP3-deficient gingival fibroblasts, *E. gingivalis* strongly activated collagenases. *E. gingivalis* effects were more pathogenic than those of the oral anaerobic bacterium *Porphyromonas gingivalis. E. gingivalis* exploits VAMP8/3-driven exocytosis pathways, driving inflammation and tissue destruction, underscoring its role as a significant periodontal pathogen.

## INTRODUCTION

It is assumed that periodontitis is caused by a polymicrobial infection in conjunction with a genetic predisposition (1). It is characterized by recurrent inflammation of the mucosa with bleeding gums and tissue damage (2–4). *Entamoeba gingivalis* is a protozoan that colonizes the oral cavity and is very common in actively inflamed periodontal pockets of cases with periodontitis (5, 6). We previously showed that *E. gingivalis* can invade the oral mucosa and kill cells there (5, 7). Infection of gingival epithelial cells (gECs) *in vitro* leads to significant activation of pro-inflammatory cytokines (7), and after antibiotic treatment, we showed the formation of protective cysts (8). This cyst formation could facilitate the persistence and infection of *E. gingivalis* and make it resistant to periodontal antibiotic treatment, suggesting resistance to certain therapies.

In genome-wide association studies, we found genetic variants in the vesicle-associated membrane protein genes *VAMP3* and *VAMP8* to be associated with periodontitis (9, 10). In the intestinal mucosa, these genes have a well-documented function in immune evasion and host defense mechanisms, respectively, of the eukaryotic parasite *Entamoeba histolytica.* In the colon epithelium, VAMP8 regulates mucin exocytosis and the formation of the mucus barrier, which keeps microorganisms at a physical distance from epithelium cells (11). *E. gingivalis* also increases mucin 21 (MUC21) expression in gingival epithelial cells (gECs) (5), a membrane-bound mucin that protects against external microbial influences. In particular, MUC21 is upregulated during gingival wound healing (12), as the regenerating tissue within a septic environment requires special protection.

Tissue invasion of the mucosa by *Entamoeba* requires collagen fiber remodeling (13). In the intestinal extracellular matrix (ECM) of the human host, *E. histolytica* promotes the overexpression and activation of human matrix metalloproteinases (MMPs), which disrupt ECM formation (14). This is dependent on the trafficking and secretion of MMPs, which degrade the ECM in connective tissue (15). MMPs are activated by *Entamoeba* cysteine proteinases such as CP-A5 from *E. histolytica* (14), and in the cells of the colon, it was shown that VAMP3 regulates MMP secretion, the inhibition of which prevents the degradation of collagen and the penetration of amoebae into the intestinal tissue (16). Additionally, MMPs are induced by pro-inflammatory cytokines, such as IL-1B (17). It was previously shown that *E. gingivalis* infection also induces significant upregulation of MMP3 and MMP13 in gingival fibroblasts (gFBs) (5).

Since symptoms of periodontitis like bleeding and barrier tissue destruction are similar to those caused by amoebiasis in the intestines and the periodontitis susceptibility genes *VAMP3* and *VAMP8* play a key role in host*–E. histolytica* interaction, in this study, we investigated the role of these genes in the interaction between *E. gingivalis* and human gECs and gFBs. To this end, we generated cell lines with loss of function mutations for *VAMP8* in immortalized lines of gECs (gEC-VAMP8(−/−) cells) and for *VAMP3* in immortalized lines of gFBs (gFB-VAMP3(−/−)), and investigated the VAMP8-dependent human immune defense and VAMP3-dependent tissue invasion mechanisms of *E. gingivalis*.

## RESULTS

### Generation of gingival *VAMP8* and *VAMP3* knockout cell lines

We created loss-of-function mutations within the protein-coding sequences of the genes *VAMP8* and *VAMP3* in gECs, cultured from the immortalized cell line of oral keratinocytes of the gingiva (OKG4) and in immortalized human gFBs (ihGF-hTERT, ABM), using the CRISPR-Cas9 system and several sgRNAs (Table S3). The designed sgRNAs targeted *VAMP8* exon 2 and *VAMP3* exons 2 and 3. We used lentiviruses (Applied Biological Materials, ABM, USA) for the transfection of OKG4 and ihGF cells with the CRISPR-Cas9 plasmids. Sanger sequencing analysis for the selected Lenti-Cas9/VAMP8-sgRNA1-infected OKG4 monoclone showed a deletion of 16 base pairs (bp) in one chromosome and a 17 bp deletion in the homologous chromosome. In the Lenti-Cas9/VAMP8-sgRNA2-infected OKG4 monoclone, a deletion of 2 bp in one chromosome and a deletion of 23 bp in the homologous chromosome were determined, confirming homozygous *VAMP8* gene knockout (Fig. S1). For the selected Lenti-Cas9/VAMP8-sgRNA3-infected OKG4 monoclone, the sequencing analysis unveiled a 4 bp deletion in allele 1 and a point mutation from cytosine (C) to thymine (T), accompanied by a 1 bp deletion in allele 2. From the Lenti-Cas9/VAMP8-sgRNA1 + 2 + 3-infected OKG4 monoclones, we found a homozygous deletion of 105 bp located between the Protospacer Adjacent Motif (PAM) sequences of sgRNA1 and sgRNA3. The mutations each led to a homozygous VAMP8 loss-of-function in the particular clones.

From the Lenti-Cas9/VAMP3-sgRNA1 + 2 + 3-infected ihGF monoclones, we selected a homozygous deletion of 3,835 bp between sgRNA1 and sgRNA3 (ihGF monoclone 1), and a homozygous deletion of 3,826 bp between sgRNA1 and sgRNA3 (ihGF monoclone 2) (Fig. S2).

For the experiments, we used OKG4 monoclone Lenti-Cas9/VAMP8-sgRNA3, named gEC-VAMP8(−/−), and Lenti-Cas9/VAMP3-sgRNA1 + 2 + 3 monoclone 1, named gFB-VAMP3(−/−).

### VAMP8 and VAMP3 localize with mucins and MMPs in gingival cells

**Fig 1:**
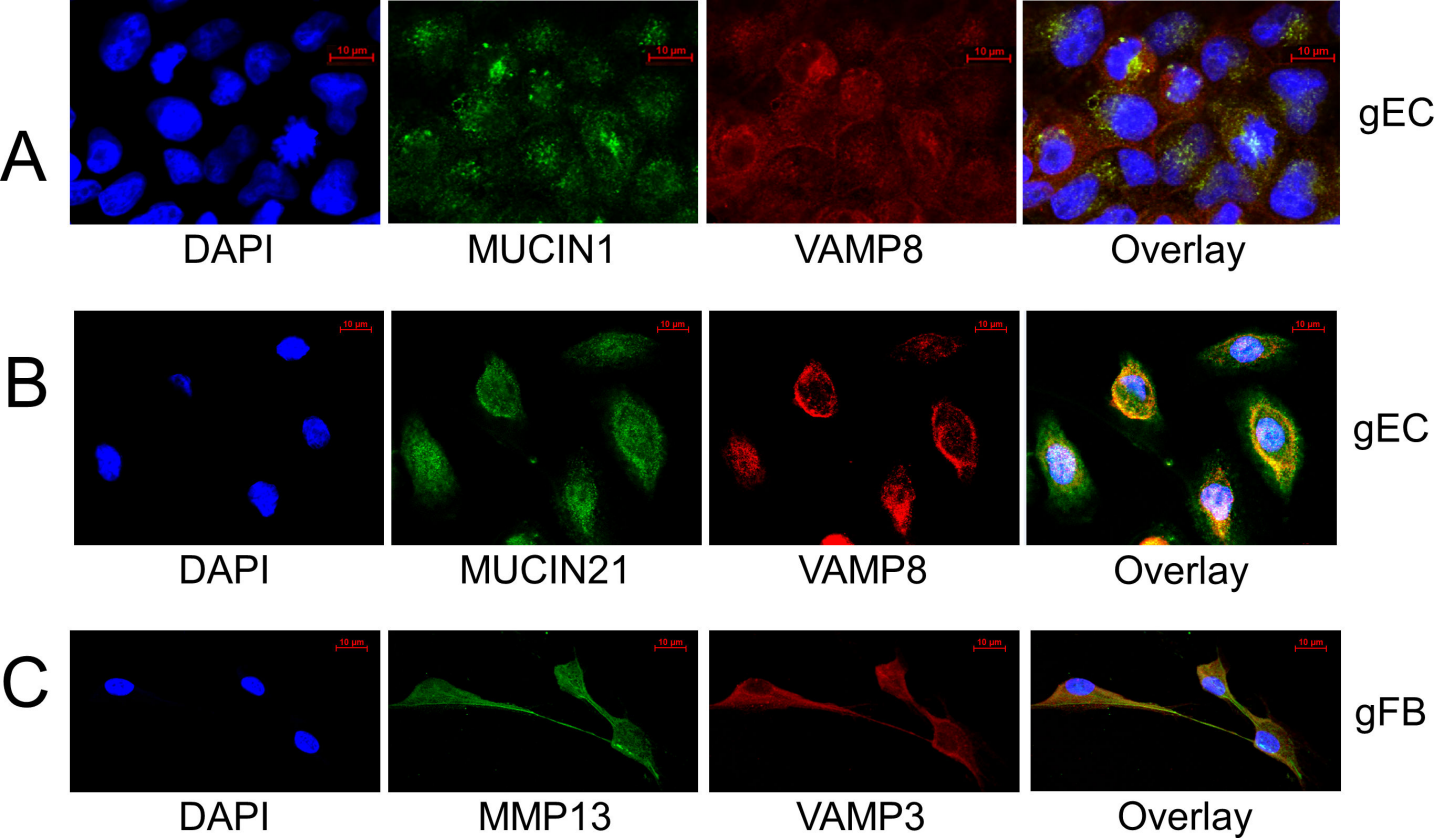
Protein co-localization in gingival cells. Cell cultures of gECs (A, B) and gFBs (C). After fixation, gingival epithelial cells were gECs, which were incubated and stained with an anti-VAMP8 antibody and (A) with an anti-MUCIN1 antibody conjugated with Alexa Fluor 546 or (B) with an anti-MUC21 antibody. In (C), gFBs were incubated and stained with an anti-MMP13 antibody and with an anti-VAMP3 antibody. Cell nuclei were stained with DAPI (blue). Co-localization was observed via fluorescent microscopy.

Based on the role of VAMP8 in mucin secretion in response to *E. histolytica* infection of epithelial cells of the colon, we hypothesized that VAMP8 would also regulate mucin secretion in gingival epithelial cells. This would require co-localization of the proteins VAMP8 with the proteins MUC1 and MUC21. To assess this, we immunostained gECs with fluorescent VAMP8, MUC1, and MUC21 antibodies. This showed co-localization of VAMP8 with both MUC1 and MUC21 in gECs (Fig. 1). We also assessed whether VAMP3 would co-localize with MMPs in gingival fibroblasts because in fibroblast cells of the colon, secretion of MMPs into the ECM depends on the function of VAMP3, and additionally, *E. histolytica* invasion of the ECM of the intestines stimulates MMP-1 and MMP-3 expression and secretion. In our experiments, immunostaining of gFBs with fluorescent antibodies for VAMP3 and MMP13 demonstrated co-localization of these proteins in gFBs (Fig. 1).

### Mucin exocytosis in gingival epithelial cells is stimulated by *E. gingivalis* infection and is mediated by VAMP8

**Fig 2:**
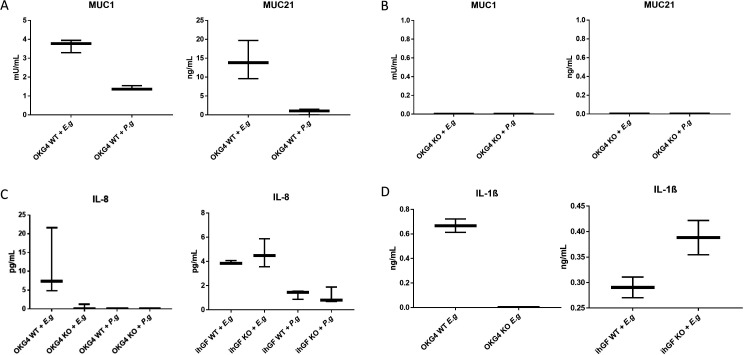
Mucin and interleukin secretion after infection of gECs and gFBs with *E. gingivalis* and *P. gingivalis*. (A) Two hours after infection of parental gECs with *E.g.*, MUC1 and MUC21 secretion was increased 3.67-fold (mU/mL; *P* = 0.001) and 14.37-fold (ng/mL; *P* = 0.0031), respectively, compared with mucin secretion in mock-infected gECs. In gEC cells that were infected with *P. gingivalis* for 2 h, no significantly increased mucin secretion was observed. (B) In gEC-VAMP8(−/−) cells, secretion of MUC1 and MUC21 was not detectable. (C) IL-8 secretion was significantly increased in parental gECs after infection with *E. gingivalis*, but could not be detected in gECs-VAMP8(−/−) cells or in gECs infected with *P. gingivalis*. IL-8 secretion was similar in both parental gFB cultures and gFB-VAMP3(−/−), indicating that VAMP8, but not VAMP3, is involved in IL-8 secretion. An infection with *E. gingivalis* caused a stronger IL-8 secretion than an infection with *P. gingivalis*. (D) IL-1B secretion was significantly increased in parental gECs cultures after infection with *E. gingivalis*, but could not be detected in gEC-VAMP8(−/−) cells. In contrast, gFB-VAMP3(−/−) cells secreted IL1B, indicating that VAMP8, but not VAMP3, is involved in IL-1B secretion. The results of A-C were obtained by spectrometry, while the results of D were obtained by chemiluminescence detection. The dark turbidity of the *P. gingivalis* growth medium prevented an experiment with mock infections using chemiluminescence detection.

During an infection with *E. histolytica*, human intestinal epithelial cells respond to the degradation of mucin, caused by the amoeba, with VAMP8-mediated hypersecretion of MUC2 (18). We hypothesized that, in response to *E. gingivalis* infection of the oral mucosa, gECs would also respond with increased VAMP8-mediated mucin exocytosis. We infected gECs cell cultures with *E. gingivalis* for 2 h. In wild-type gECs, *E. gingivalis* infection induced MUC1 and MUC21 secretion 3.67-fold (mU/mL; *P* = 0.001) and 14.37-fold (ng/mL; *P* = 0.0031) compared with wild-type gECs-controls, which were mock-infected with the supernatant from the last of three PBS wash steps of *E. gingivalis* cultures (Fig. 2). In contrast, the secretion of MUC1 and MUC21 was not detectable in gEC-*VAMP8*(−/−) cells.

To compare the effect of *E. gingivalis* with that of a bacterial strain, which like *E. gingivalis* commonly colonizes periodontal anaerobic pockets, we also infected gECs with the bacterium *P.g*. We observed that *P. gingivalis* infection for 2 h had no significant effect on MUC1 (1.42 (mU/mL; *P* = n.s.) and on MUC21 secretion (1.27-fold increase ng/mL; *P* = n.s.).

In gEC-*VAMP8*(−/−) cells, however, secretion of MUC1 and MUC21 was not detectable with or without *E. gingivalis* and *P. gingivalis* infection, demonstrating that mucin secretion in gECs is mediated by VAMP8.

### IL-8 and IL-1B secretion by gingival epithelial cells is enhanced by infection with *E.g*., but is blocked in *VAMP8*(−/−) cells

We showed in an earlier study that the expression of *IL-8* is strongly increased after *E. gingivalis* infection of gECs (7). Here, we investigated whether IL-8 protein secretion would also be increased after infection with *E. gingivalis* and whether VAMP8 is involved in IL-8 exocytosis. After 2 h of infection with *E.g*., we observed a 7.4-fold increase in IL-8 secretion in cultures of parental gECs compared to mock-infection (*P* = 0.019) (Fig. 2). In gEC-*VAMP8*(−/−), we could not detect IL-8 secretion, neither in mock-infected nor *E.g*.-infected cells, demonstrating that IL-8 secretion in gECs is mediated by VAMP8.

After 2 h of *P. gingivalis* infection, we found no IL-8 secretion either in parental gECs nor in gEC-VAMP8(−/−), demonstrating that *P.g* does not significantly induce IL-8 secretion in gECs.

Next, we tested how gFBs would respond to *E.g.* infection in terms of IL-8 secretion. After *E.g*. infection of cultures of parental gFBs and gFB-VAMP3(−/−) cells, we observed IL-8 secretion increased by 3.9- and 5.8-fold, respectively, compared to mock-infected cells. The similar secretion of IL-8 in parental gFB cells and VAMP3(−/−) cells indicated that loss of VAMP3, unlike the loss of VAMP8, did not affect IL-8 secretion.

After *P.g*. infection of cultures of parental gFBs and gFB-VAMP3(−/−) cells, we observed low IL-8 secretion increased by 1.3-fold and 1.4-fold, respectively, compared to mock-infected cells (*P* = n.s.).

Since we found that the function of VAMP8 is required for the secretion of IL-8, which is particularly produced by epithelial cells and fibroblasts to attract neutrophils in response to infection, we also tested whether VAMP8 was required for the secretion of interleukin 1B (IL1B). This cytokine causes a broader activation of the innate immune system. Following infection with *E.g*., we observed an increase in IL-1B secretion from parental gECs cell cultures (0.6 ng/mL) compared to mock-infected cells. In gEC-VAMP8(−/−), we detected no IL-1B secretion.

Following infection of gFBs with *E.g*., we detected a moderate increase in IL-1B secretion in parental gFBs (0,27 ng/mL) as well as in gFB-VAMP3(−/−) (0.35 ng/mL), compared to mock-infected cells. The results show that gECs secrete more IL-1B after infection with *E. gingivalis* compared to gFBs and that VAMP8, but not VAMP3, regulates IL-1B secretion.

### *E. gingivalis* infection induces VAMP8 translocation into the cell membrane

**Fig 3:**
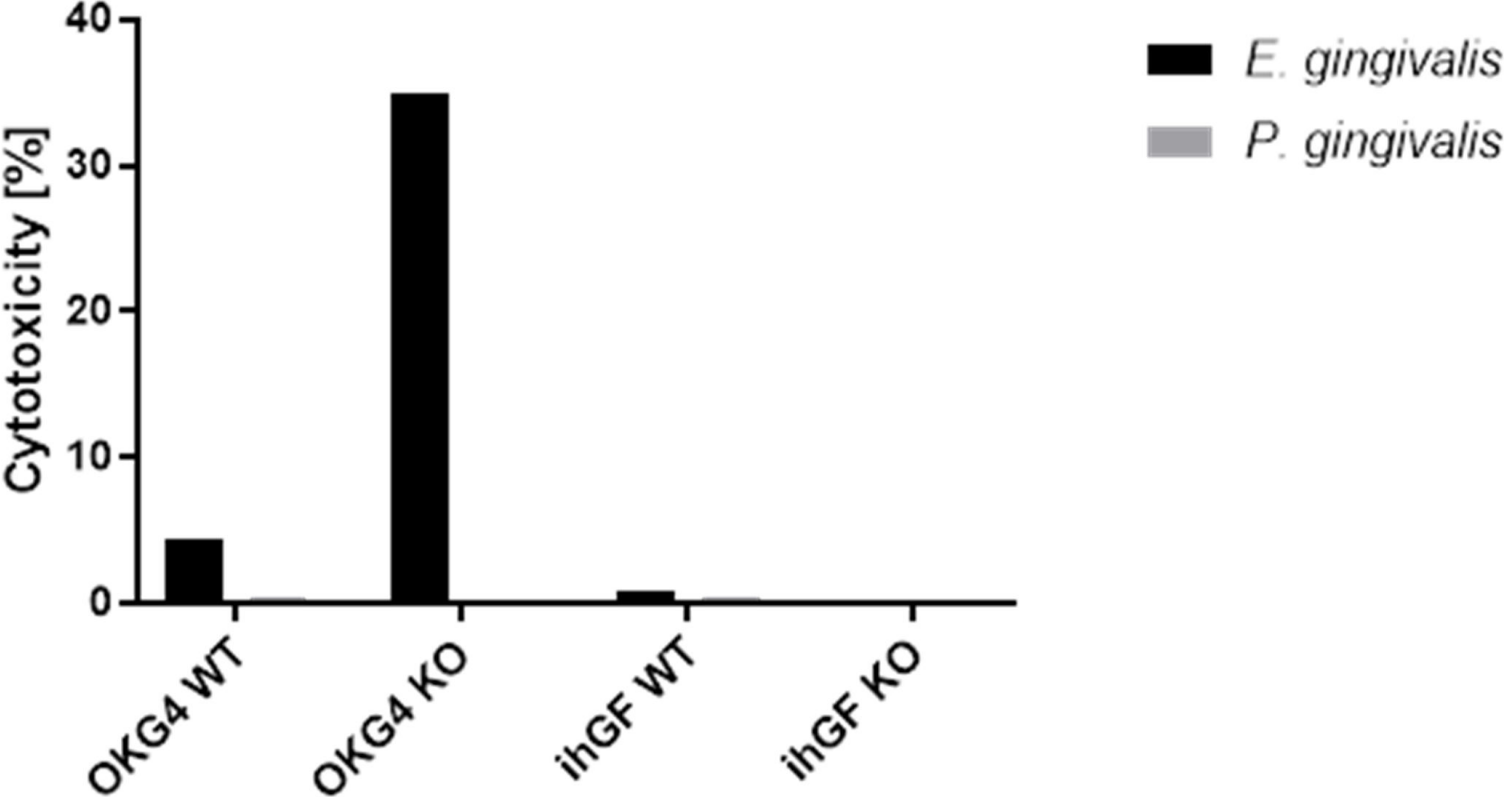
VAMP8 loss of function leads to increased cell death in gECs after infection with *E. gingivalis.* A two-hour infection with *E. gingivalis* increased cell death by 4% in the parental gECs and by 35% in the gEC-VAMP8(−/−) lines. In both the parental gFBs and the gFBs-VAMP3(−/−) cell lines, infection with *E. gingivalis* did not lead to an increase in the cell death rate compared to mock-infected cells. A 2 hour infection with *P. gingivalis* did not lead to increased cell death in any of the cell lines.

VAMP8 does not normally associate with the cell membrane as it is localized in the cytoplasm, but during exocytosis of vesicles, it translocates into specialized microdomains within the semi-permeable lipid bilayer of the cell membrane, which are termed lipid rafts and serve as platforms for cellular processes by concentrating proteins. Lipid rafts are involved in exocytosis, and VAMP8 present here after secretion is a readout for participation in exocytosis. To confirm translocation of VAMP8 into the cell membranes following *E. gingivalis* infection, we isolated lipid raft domains of the cell membranes from *E.g*.-infected and mock-infected gEC- and gEC-VAMP8(−/−) lines. gECs grown under normal culture conditions had little VAMP8 present within lipid raft domains (Fig. 3). However, after infection of gECs with *E.g*., we observed a significantly stronger VAMP8-antibody stained band, indicating increased translocation of VAMP8 into lipid raft domains of the cell membrane. The observed VAMP8-antibody stained band runs at 50–75 kDa on the gel, which is larger than the expected size of the pure VAMP8 protein (~25 kDA). This indicated a possible integration into larger protein-lipid microdomains or multimerization.

In gEC-VAMP8 (−/−) cells, however, the association of VAMP8 with the plasma membrane was not observed. This indicated that VAMP8 participates in exocytosis of gECs after *E. gingivalis* infection.

### *E. gingivalis* infection triggers gEC cell death, exacerbated by VAMP8 deficiency

**Fig 4:**
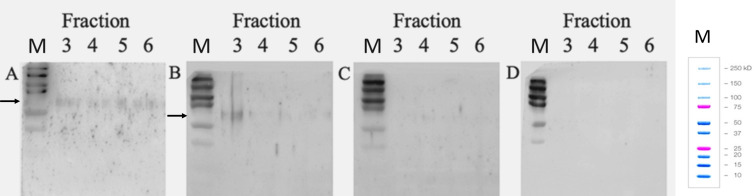
Western blot with anti-VAMP8 antibody of gEC protein fractions after isolation of lipid rafts by ultracentrifugation Protein fractions from (A) parental gECs without *E. gingivalis* infection, (B) parental gECs after *E. gingivalis* infection for 2 h, (C) gEC-VAMP8(−/−) cells without E. gingivalis infection and (D) gEC-VAMP8(−/−) cells after *E. gingivalis* infection for 2 h. M = Protein size marker. A weak VAMP8-antibody stained band is seen in parental gECs without *E. gingivalis* infection, which is significantly stronger after *E. gingivalis* infection, indicating increased translocation of VAMP8 into the cell membrane. The observed VAMP8-antibody stained band is seen at 50–75 kDa on the gel, which is larger than the expected size of the pure VAMP8 protein (~25 kDA), indicating integration into the larger protein-lipid microdomain. Fractions 3 and 4 correspond to 5% sucrose after ultracentrifugation, and fractions 5 and 6 correspond to 40% sucrose, see Supplemental Material for details.

In our previous studies, we showed that *E. gingivalis* kills gECs by lysing pores into the cell membranes and taking up the chromatin of the host cells. Mucin is the main component of the protective mucus barrier of mucosal tissues, including the oral mucosa, and forms a biochemical shield between the barrier tissue and the biofilm. In the colon, as an important protective mechanism against infection, mucin prevents direct contact between *E.h*. and epithelial cells. To prove the protective effect of mucin against *E.g*., we investigated whether the loss of mucin secretion by *VAMP8* knockout increases the death of gECs after infection. Following infection of parental gECs and gEC-VAMP8(−/−) lines with *E. gingivalis* for 2 h, we observed a 3.6-fold and 40.3-fold increase in cell death markers, respectively, compared to mock infection (Fig. 4).

We also investigated the cytotoxicity of *E. gingivalis* infection on gFBs. We found that infection with *E. gingivalis* did not significantly increase cell death of gingival fibroblasts, neither in parental gFBs (1.5-fold) nor gFB-VAMP3(−/−) lines (1.8-fold).

To compare the effect of *E. gingivalis* with that of infection with the oral bacterium *P.g*., we infected parental gECs and gFBs as well as gEC-VAMP8(−/−) and gFB-VAMP3(−/−) lines with *P.g*. In parental and gEC-VAMP8(−/−), we observed no increase in cell death after *P. gingivalis* infection for 2 h. In parental cell cultures of gFBs, we observed a 2.1-fold increase in cell death after *P. gingivalis* infection for 2 h, compared to mock infection. In gFB-VAMP3(−/−) cells, we observed a 3.7-fold increase in cell death markers compared to mock-infected gFB-VAMP3(−/−) cells. This showed that *E. gingivalis* infection, but not *P. gingivalis* infection, triggers cell death in gECs and that cell death of epithelial cells in response to *E. gingivalis* infection is enhanced by VAMP8 deficiency. However, gFBs are not killed by *E. gingivalis*

### Collagenase activation in gingival fibroblasts is driven by E. gingivalis and blocked in gFB-VAMP3(−/−) cells

**Fig 5:**
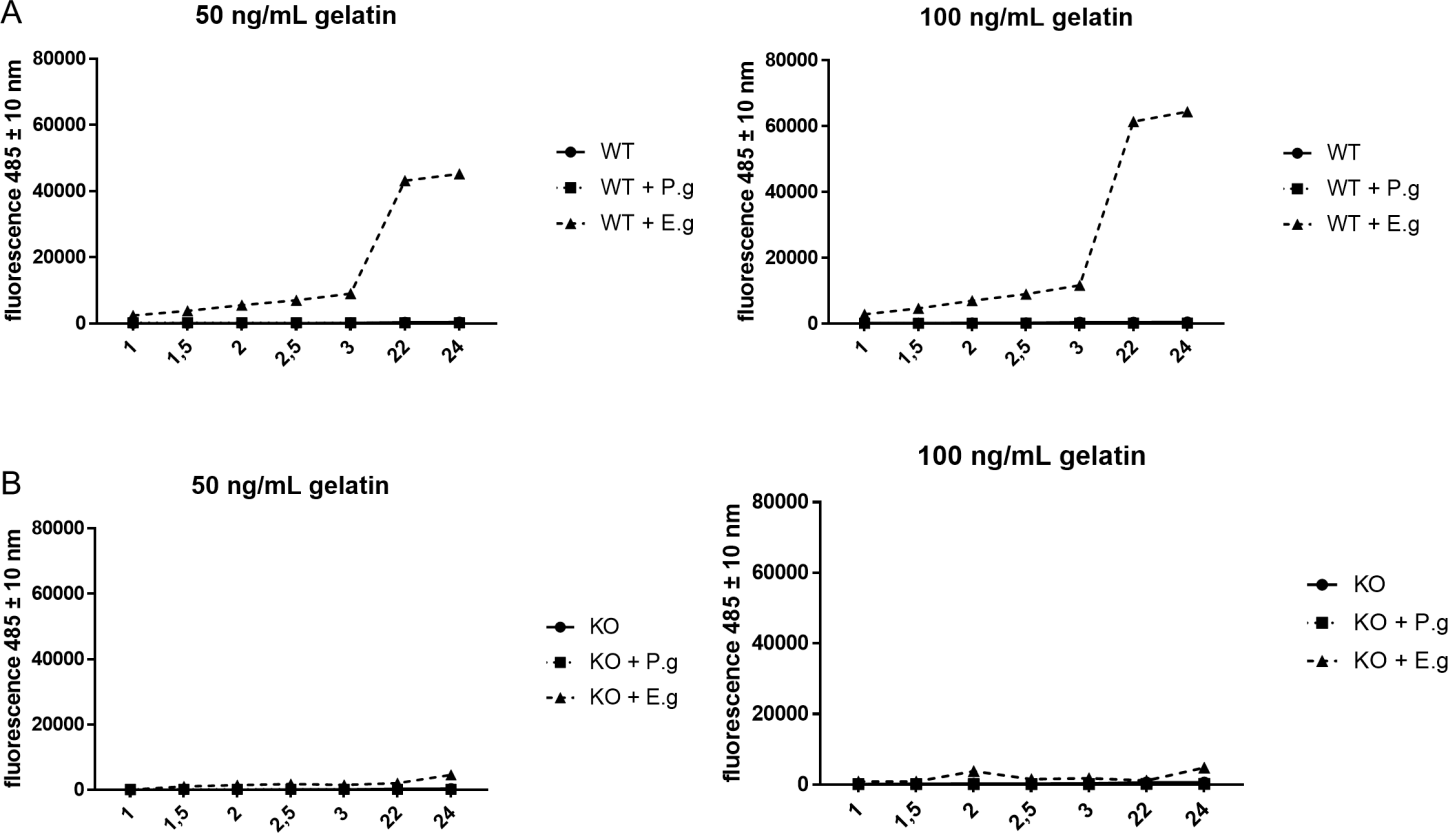
MMP-dependent collagen degradation was induced in gFBs by infection with *E. gingivalis*. (A) Infection with E. gingivalis caused gelatin degradation in parental gFBs. Gelatin degradation was not observed without *E. gingivalis* infection or after infection with the oral bacterium *P. gingivalis*. (B) In gFB-VAMP3(−/−) cells, infection with *E. gingivalis* did not lead to gelatin degradation, indicating that VAMP3 is required for MMP secretion. For the collagenase assay, the media supernatant of gFBs was in combination with different gelatinase concentrations (50 ng/mL and 100 ng/mL).

We investigated the role of VAMP3 in collagen degradation in response to *E. gingivalis* infection. gFBs cultured under normal growth conditions exhibited no collagenase activity. However, after 3 h of infection with *E. gingivalis* trophozoites, we observed a strong induction of collagenase activity, which was not seen after infection with *P. gingivalis* (Fig. 5). In gFB-VAMP3(−/−) lines, however, we did not observe collagenase activity with or without *E. gingivalis* or *P. gingivalis* infection. This showed that VAMP3 is required for MMP secretion and collagen cleavage in gingival fibroblasts and that *E. gingivalis* induces secretion and activation of host collagenases.

## DISCUSSION

The results of our study show that *E. gingivalis* triggers mechanisms of immune defense and tissue degeneration in gingival cells that exhibit similar patterns to infections with *E.h*. in the intestines. The study provides evidence that the vesicle-associated membrane proteins VAMP8 and VAMP3 play a central role in the mucosal defense mechanism of the gingiva and in the pathogenetic activity of *E. gingivalis* during infection. Overall, these findings support the hypothesis that VAMP8 and VAMP3 are important proteins in the modulation of oral host defense mechanisms and tissue destruction in periodontitis-associated infections.

The results also show that impaired function of VAMP8 significantly weakens the protection of the gingival barrier by reducing mucus secretion, presumably facilitating or allowing direct contact of *E. gingivalis* with the oral epithelial cells, enabling *E. gingivalis* to attack and kill the epithelial cells. We also showed that MMP secretion in fibroblasts of the oral connective tissue is mediated by VAMP3, which is utilized by *E. gingivalis* for tissue destruction by inducing MMP secretion and activation, allowing the degradation of collagen fibers.

Our findings also demonstrate the specific host tissue defense response that is induced by *E. gingivalis* infection, which is mediated in part by the activity of VAMP proteins. It was shown that VAMP8 plays an essential role in mucosal defense against *E.h*. in the gut by promoting the secretion of MUC2 from goblet cells, thereby maintaining a physical barrier against the pathogen (11). Similarly, in the current study, we found that infection of gingival epithelial cells by *E. gingivalis* leads to a strong secretion of the gingiva-expressed MUC1 and MUC21, and that this is dependent on VAMP8 activity. This process presumably contributes to the integrity of the oral mucosa and prevents direct interaction of the amoebae with the epithelial cells. In contrast, infection with *P. gingivalis* did not result in significant mucin secretion, suggesting that the mucosal defense mechanism is not tuned to *P. gingivalis* but specifically tuned to *E. gingivalis* and microbes of similar pathogenicity.

The importance of VAMP3 in the secretion of MMPs in fibroblasts of the oral connective tissue confirms previous findings on its role in the gut. Previous studies showed that VAMP3 is crucial for the secretion of MMPs required for intestinal ECM degradation (14). Our results suggest that these mechanisms also play a role during periodontal infection by *E. gingivalis* and contribute to tissue degradation, particularly in the induction of MMP13. The collagenase activity that we observed exclusively in *E. gingivalis*-infected gFBs but not in gFB-VAMP3(−/−) cells supports the notion that VAMP3 is necessary for MMP-mediated ECM degradation in the gingival ECM and that VAMP3-dependent MMP secretion responds to pathogenic infections. By knocking out *VAMP3*, the secretion of these MMPs and thus collagen degradation was completely blocked, demonstrating a central role of this protein in the invasive mechanisms during E. gingivalis infections.

The study has some limitations. First, the experiments were conducted under *in vitro* conditions, which may limit the relevance to the *in vivo* situation. Second, our study is limited to analyzing the early infection phase. Long-term interactions between the host and *E.g*., which may have a stronger influence on chronic inflammatory processes and tissue remodeling processes, were not investigated. Finally, further studies on the function of VAMP8 and VAMP3 in other cells of periodontal tissue would be desirable, as these could potentially provide additional insights into the overall function of these proteins in periodontitis-associated immune defense.

The results of this study confirm the role of VAMP8 and VAMP3 as central regulators in the immune defense of the gingiva against *E.g*. The targeted induction of MUC1 and MUC21 by VAMP8 and the VAMP3-mediated secretion of MMPs indicate that *E. gingivalis* is able to breach the mucosal defense barriers and damage gingival connective tissue. We conclude that VAMP8 and VAMP3 may be potential targets for therapeutic approaches to limit tissue destruction in periodontitis-associated infections and protect mucosal integrity. The results also imply that *E. gingivalis* is a serious oral pathogen contributing to chronic inflammation and tissue destruction.

## MATERIALS AND METHODS

### Generation of gingival *VAMP8* and *VAMP3* knockout cell lines

We generated functional knockouts of *VAMP8* in gECs cultured from immortalized oral keratinocytes of the gingiva (OKG4, obtained from the Harvard Skin Disease Research Center Cell Culture Lab, Boston, MA, USA Jim Rheinwald), named gEC-VAMP8(−/−), and of *VAMP3* in gFBs cultured from immortalized human gingival fibroblasts (ihGF h-TERT, ABM), named gFB-VAMP3(−/−), using CRISPR-Cas9 as described in the Supplemental Methods.

### *E. gingivalis* collection and handling

Subgingival plaque samples were collected with sterile curettes from actively inflamed periodontal pockets (bleeding on probing) of patients who had been diagnosed with periodontitis at the Department of Periodontology, Charité—Universitätsmedizin Berlin, Germany. The subgingival plaque samples were transferred to TYGM-9 medium and kept under anaerobic conditions in Petri dishes at 35°C for up to 2 days. The presence of *E. gingivalis* trophozoites was analysed under the microscope. Prior to infection, the Petri dishes containing *E. gingivalis* were placed on ice for 3´ to detach the amoebae from the bottom of the dish. The amoeba were washed in PBS by centrifugation (300 g, 5´, 3×). The washed supernatant was used for mock infection.

The plaque donors’ rights have been protected by the Institutional Review Board (ethical approval number EA1/169/20) and written informed consent was given by the subjects.

### *Porphyromonas gingivalis* cultivation

To compare the pathogenicity of *E. gingivalis* with an oral bacterium described as pathogenic, which frequently colonizes anaerobic periodontal pockets, we used *Porphyromonas gingivalis* (*P. gingivalis*, strain W83). This strain was obtained from the Leibniz Institute DSMZ-German Collection of Microorganisms and Cell Cultures, Braunschweig, Germany, and grown on Columbia blood agar plates (100 µL cell suspension) in an anaerobic container at 37°C for 4–7 days. Before use, live *P. gingivalis* colonies were scraped off with an inoculation loop and resuspended in PBS. The living bacterial suspensions were then added to human cell cultures (MOI = 5) in order to quantify the pathogenic effects. As a negative control for these experiments, we heat-inactivated *P. gingivalis* suspensions at 95°C for 5´ and then inoculated the cells with the killed bacterial suspension (MOI = 5).

### Gingival cell culture

ihGF h-TERT (gFBs) and gFB-VAMP3(−/−) were grown in Dulbecco’s modified Eagle’s medium (DMEM, PAN Biotech, Germany) supplemented with 10% fetal bovine serum (FBS, Gibco, USA) and 1% non-essential amino acids (MEM-NEAA, PAN Biotech) at 37°C and 5% CO_2_ as described before (5). OKG4/bmi1/TERT (gECs) and gEC-VAMP8(−/−) were grown in DermaLife K complete medium (LIFELINE Cell Technology).

### Protein co-localization by immunofluorescence

Gingival cells were cultured on coverslips. After fixation, the cells were incubated with fluorescently labeled antibodies (VAMP8, −3, MUC2, −21, and MMP13) and stained. Detailed information on the antibodies and staining methods is described in the Supplemental Material.

### IL-8, MUC1, and MUC21 Enzyme-linked Immunosorbent Assays (ELISA)

Parental (wildtype) gECs and gECs-VAMP8(−/−) cells were grown to 90–95% confluence. Cells were infected with *E. gingivalis* (MOI = 0.0004) or *P. gingivalis* (MOI = 5) for 2 hrs. ELISAs were performed according to the manufacturer’s protocols using ELISA Kits for human MUC1- (EHMUC1, ThermoFisher), MUC21 (HUFI00559, AssayGenie) and IL-8/CXCL8 (Sigma-Aldrich; RAB0319) on a microplate spectrophotometer (Multiskan GO, Thermo Fisher).

### IL-1B immunoassay

Parental gECs and gEC-VAMP8(−/−) cells were grown to 90–95% confluence. Cells were infected with *E. gingivalis* (MOI = 0.0004) or *P. gingivalis* (MOI = 5) for 2 hrs. Immunoassay was performed according to the manufacturer’s protocol using Lumit IL-1β Human/Mouse Immunoassay (W6010, Promega). Released IL-1β in cell culture samples was quantitatively measured on a microplate luminometer (GloMax Explorer Multimode Microplate Reader, Promega).

### Lipid raft isolation, immunoblotting, cytotoxicity, and collagenase assays

A detailed description of lipid raft isolation and immunoblotting and methods used for quantification of cell death rate and collagenase activity are described in the Supplemental Methods.

## SUPPLEMENTAL MATERIAL

Supplemental material iai.00005-25-s0001.pdf Fig. S1 to S4; Tables S1 and S2.

ASM does not own the copyrights to Supplemental Material that may be linked to, or accessed through, an article. The authors have granted ASM a non-exclusive, world-wide license to publish the Supplemental Material files. Please contact the corresponding author directly for reuse.
