# Supplementary material for: Entamoeba gingivalis induces gingival cell death, collagen breakdown, and host immune response via VAMP8/-3-driven exocytosis pathways
Source: Infect Immun. 2025 Mar 21;93(4):e00005-25. doi: 10.1128/iai.00005-25 (PMC11977317; doi:10.1128/iai.00005-25)
Supplement: Supplemental material — Fig. S1 to S4; Tables S1 and S2. [file iai.00005-25-s0001.pdf]

Appendix methods

Generation of gingival *VAMP8* and *VAMP3* knockout cell lines

To generate functional knockouts of *VAMP8* in gingival keratinocytes (gECs; cell line OKG4) and of *VAMP3* in human gingival fibroblasts (gFBs, cell line gFB-hTERT; ABM) using CRISPR-Cas9, sgRNAs were designed to target early exons of VAMP3 and VAMP8 using the sgRNA design tool 'CRISP-ERA' (http://crispr-era.stanford.edu) and the 'gene editing using nuclease' algorithm implemented in this software tool, which finds the sgRNAs in exon region (http://crispr-era.stanford.edu/next.jsp#s2). sgRNAs were designed to target early exons. Each target sequence was calculated for 2 scores: the efficacy score (E score) and the specificity score (S score). The selected sgRNA sequences were cloned into the vector pLenti-U6-sgRNASFFV-Cas9-2A-Puro (Applied Biological Materials; ABM) and lentiviruses were cultured in HEK293 cells. The sgRNA sequences are shown in **Appendix Figures 1 and 2**. We used lentiviruses that contained sgRNAs, which were cloned into pLenti-U6-sgRNASFFV-Cas9-2A-Puro, to infect immortalized human gingival fibroblasts and immortalized gECs (obtained from the Harvard Skin Disease Research Center Cell Culture Lab, Boston, MA, USA Jim Rheinwald).

```
              *         *         *         *
1>GAAGGTGGAGGAAATGATCGTGTGCGGAACCTGCAAAGTGAGGTGGA>47
1>GAAGGTGGA----------------GGAACCTGCAAAGTGAGGTGGA>31
1>GAAGGTG-----------------CGGAACCTGCAAAGTGAGGTGGA>30

Allele 1 - Matches:31; Mismatches:0; Gaps:16
Allele 2 - Matches:30; Mismatches:0; Gaps:17
```

A

```
              *         *         *         *
1>AAATGATCGTGTGCGGAACCTGCAAAGTGAGGTGGAGGGAGTTAAGAAT>49
1>AAATGATCGTGTGCGGAACCTGCAAAG--AGGTGGAGGGAGTTAAGAAT>47
1>AAATGA-----------------------AGGTGGAGGGAGTTAAGAAT>26

Allele 1 - Matches:47; Mismatches:0; Gaps:2
Allele 2 - Matches:26; Mismatches:0; Gaps:23
```

B

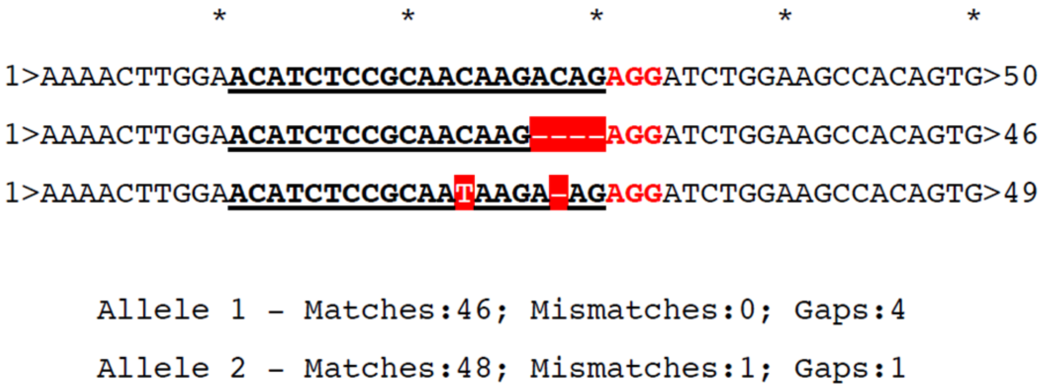

C

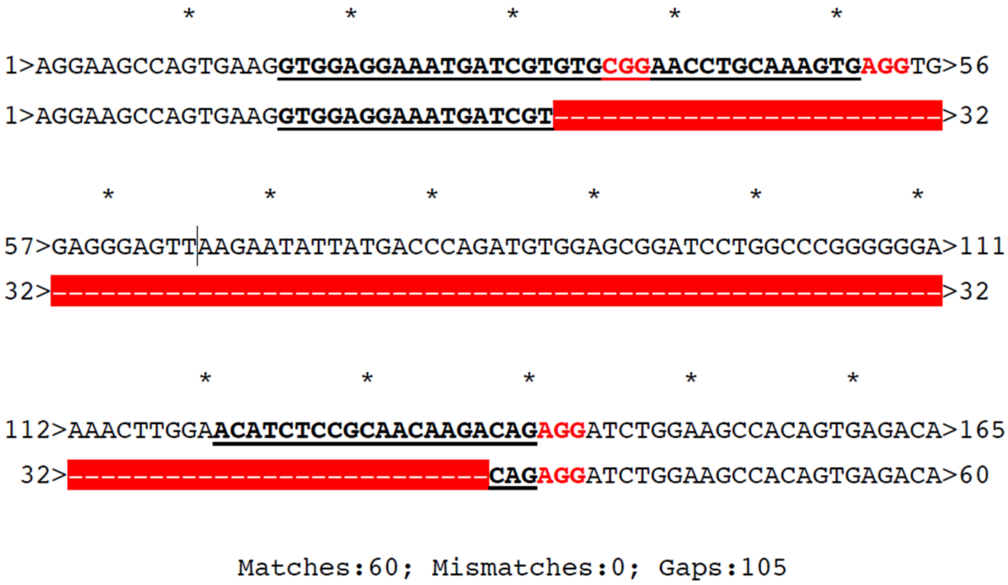

D

Appendix Figure 1. Sequencing analysis of the Lenti-Cas9/VAMP8-sgRNA1, -sgRNA2, -sgRNA3 and -sgRNA1+2+3 gEC monoclones

After analyzing the raw trace files, both possible alleles of each of the Lenti-Cas9/VAMP8-sgRNA infected gEC monoclones were manually aligned to the wild-type VAMP8 genomic sequence. The gRNAs are underlined. The corresponding PAM sequences are shown in red.

A: The sequencing analysis of the Lenti-Cas9/VAMP8-sgRNA1 infected gEC monoclone revealed two deletions of 16 and 17 bp.

B: The sequencing analysis of the Lenti-Cas9/VAMP8-sgRNA2 infected gEC monoclone reveals a deletion of 2 bp in allele 1, as well as a larger deletion of 23 bp in allele 2, confirming the successful knockout of VAMP8. A section of this alignment is shown with the sgRNA2 being underlined.

C: The sequencing analysis unveiled a 4 bp deletion in allele 1 and a point mutation from cytosine (C) to thymine (T), accompanied by a 1 bp deletion, in allele 2. The section of this alignment is shown with the sgRNA3 being underlined.

D: A homozygous deletion of 105 bp located between the PAM sequences of sgRNA1 and sgRNA3 was revealed after alignment of the Lenti-Cas9/VAMP8-sgRNA1+2+3 gEC infected monoclone with the wild-type VAMP8 genomic sequence.

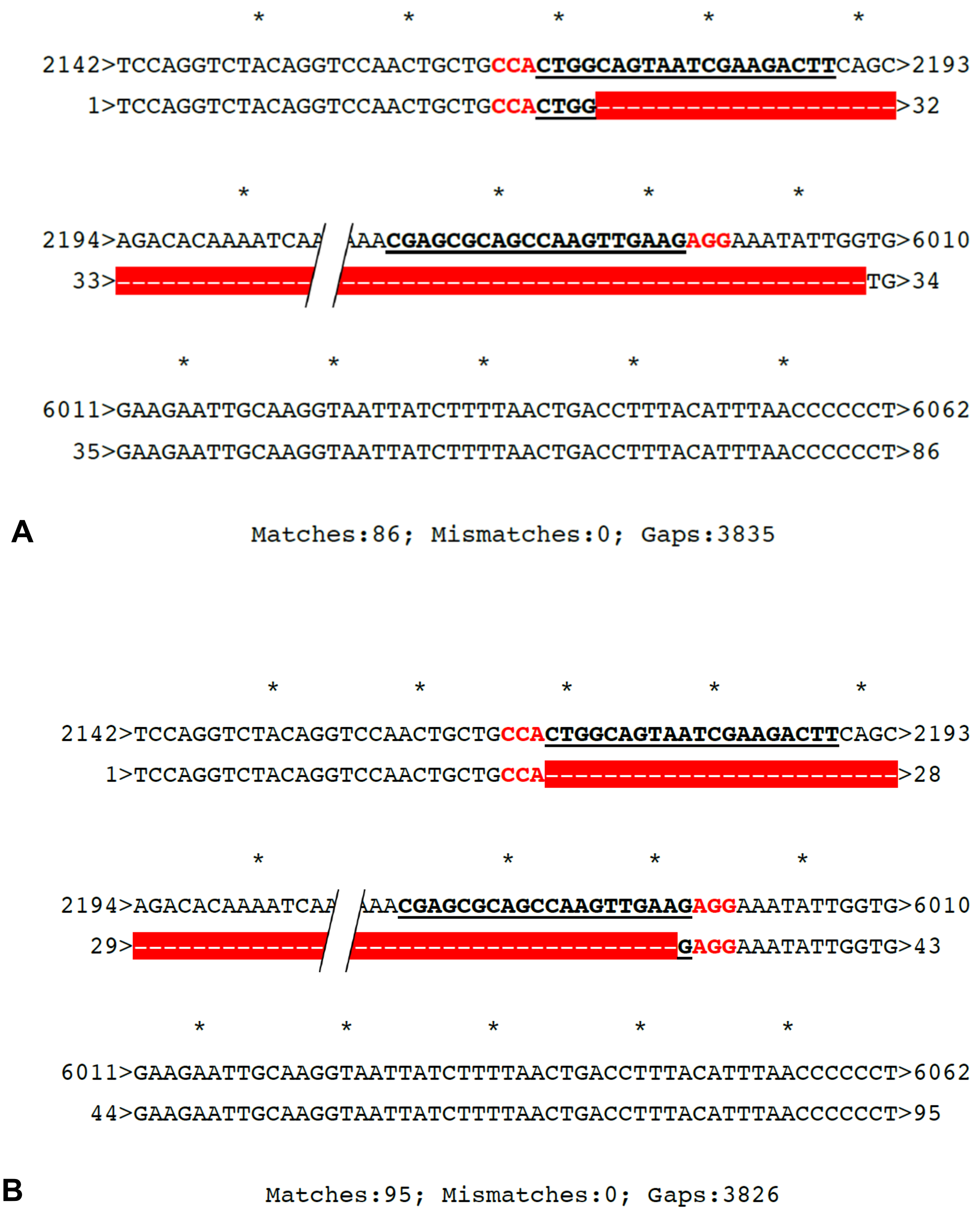

Appendix Figure 2. Sequencing analysis of the Lenti-Cas9/VAMP3-sgRNA1+2+3 gFB monoclones 1 and 2

Section of the automatic alignment using the Lenti-Cas9/VAMP3-sgRNA1+2+3 infected gFB monoclone 1 VAMP3 sequence with the wild-type VAMP3 genomic sequence. sgRNAs are underlined whereas the corresponding PAM sequences are shown in red.

A. For the gFB monoclone 1, the alignment reveals a homozygous deletion of 3835 bp between sgRNA1 and sgRNA3 (underlined).

B. The alignment of the VAMP3 sequence for the gFB monoclone 2 reveals a homozygous deletion of 3826 bp between sgRNA1 and sgRNA3 (underlined).

The 293T cells were seeded in 10 cm cell culture dishes at ~ 40-50% confluency the day before transfection. At 70-80% confluency the medium was replaced with 5 mL fresh antibiotic free cell culturing medium and, using jetPEI, the 293T cells were transfected with 10 µg pLenti-U6-sgRNASFFV-Cas9-2A-Puro and 10 µg 3rd Generation Packaging Mix. The virus titer was measured on the same day (qPCR Lentivirus Titer Kit, ABM). The virus containing supernatants were then used for in vitro infections within 48 h. gFB and gECs were seeded in 24-well plates and incubated at 37°C 24 h pre-infection, according to the guidelines (ABM). For infection, a mixture of complete medium, medium containing the lentiviruses and ViralEntryTransduction Enhancer (100X) (ABM) at a 1:100 ratio was prepared. The growth medium was removed from the wells and replaced with 500 µL of the transduction mixture, followed by a 24 h incubation at 37°C. ihGF cells were transfected with Lenti-Cas9/VAMP3-sgRNA and Lenti-GFP lentiviruses at a MOI of 70 and gECs were transfected with Lenti-Cas9/VAMP8-sgRNA and Lenti-GFP lentiviruses at a MOI of 50. A medium change was performed 24 h post-infection. In order to select for positively transduced gFBs and gECs, a puromycin selection was performed 24 h post-infection (ihGF 3 mg/ml puro, gEC 30 mg/ml puro).

Monoclonal knockout cell lines were created by limiting dilution cloning. For this purpose, 100 µL of complete medium containing the appropriate puromycin concentration were added to each well of a 96-well plate. 200 µL of initial cell suspension containing ~ 4000 cells were added to well A1 and 1:2 dilutions were made vertically according to **Appendix Figure 3**. To keep the total volume the same for all wells, 100 µL medium was removed from well H1 and an additional 100 µL medium were added to each well of column 1. Using a multichannel pipette this processed was repeated horizontally until column 12 was reached. Again, 100 µL medium were removed from each well of column 12. After both dilution series were completed, well A12 should have approximately 1 cell/well. In order to isolate monoclonal gFBs, 'easy-to-kill' primary fibroblasts, which bear no resistance gene for puromycin, had to be added to wells containing only one positively selected gFBs. For this purpose, after completing the single cell dilution procedure for gFBs, wells containing only one cell were marked, filled with approximately 100 primary fibroblasts and cultured for three days using complete medium before applying antibiotic containing medium. After approximately 1-2 weeks, individual monolayers that derived from one monoclone were transferred to a 24-well plate followed by a 6-well expansion.

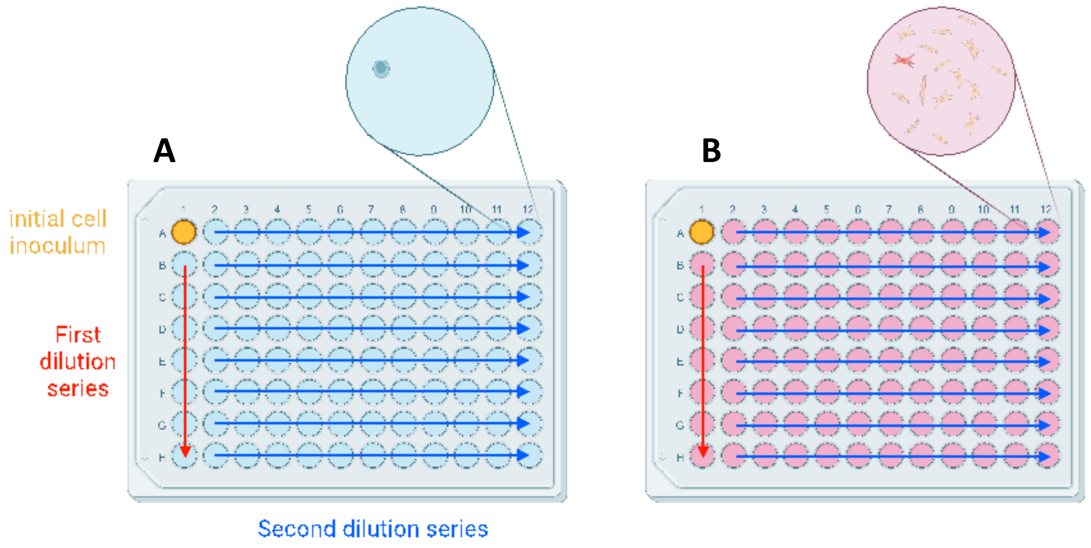

Appendix Figure 3: Illustration of first and second dilution series for monoclonal isolation of gEC and gFB cells

(A) Single cell dilution process for gECs (blue). With an initial amount of ~ 4000 cells in well A1, well A12 should have approximately 1 cell/well. **(B)** Single cell dilution process for gFB cells (red). With an initial amount of ~ 4000 cells in well A1, well A12 should have approximately 1 cell/well. After completing the serial dilution for gFB cells, the wells were filled with ~ 100 primary fibroblasts (yellow). Adapted from "CRISPR Cas9 An Introductory Guide for Gene Knockout Notice to Purchaser, figure 5: Illustration of first and second dilution series for monoclonal isolation" (Applied Biological Materials Inc.), Created with BioRender.com

Gene editing was validated by T7 Endonuclease I mutation detection (EnGen Mutation Detection Kit, New England BioLabs) and Sanger sequencing (LGC Genomics, Berlin, Germany). Primer sequences and PCR settings are shown in **Appendix Tables 1-2**.

Appendix Table 1. T7 Endonuclease I mutation detection

Cell line	Primer	sequence	Product size (bp)	Cleaved products (bp)
gFB	VAMP3-T7-A fwd	TCCAGGTCTACAGGTCCAAC	A: 232	T1: 206 + 26
	VAMP3-T7-A Rev	TAGATCTTCCAGCACAGCAG		
	VAMP3-T7-B fwd	GTGATCCACCCACCTCGG	B: 406	T2: 138 + 268<br>T3: 161 + 245
	VAMP3-T7-B Rev	GTGGACCCACATTTCCATGT		
gEC	VAMP8-T7 fwd	TCTGAGCCCAAGTCTCCAGT	A: 197	T1: 99 + 98<br>T2: 114 + 83

	VAMP8-T7-A Rev	GTTGCGGAGATGTTCCAAGT		
	VAMP8-T7 fwd	TCTGAGCCCAAGTCTCCAGT	B: 229	T3: 204 + 25
	VAMP8-T7-B Rev	TGTCTCACTGTGGCTTCCAG		

The PCR products were verified on a 2% agarose gel at 120 V in terms of the correct product size and specificity and were purified using either ethanol precipitation (4.10) or gel extraction (4.14) if too many unspecific bands were visible. The purified PCR products were then used for the detection of on-target genome editing events using T7 Endonuclease I. For this purpose, 200 ng of the respective PCR products were mixed with 2 µL 10x NEBuffer 2 and UPW to a total volume of 19 µL followed by a denaturation and re-annealing using the following conditions (Appendix Table 2). This resulted in the generation of heteroduplexes when mutations from insertions and deletions (indels) are present in the amplicon pool.

Appendix Table 2. T7 mutation detection hybridization conditions

**Reaction Step**	**Temperature (°C)**	**Ramp rate (°C/sec)**	**Time (min)**
Initial denaturation	95		5
Annealing	5-85	-2	
	85-25	-0.1	
Hold	4		∞

Protein co-localization by immunofluorescence

The cells were cultured on coverslips. After fixation, gingival epithelial cells were incubated and stained an anti-VAMP8 antibody (abcam cat. no. ab89158) and a secondary antibody conjugated with Alexa Fluor 647 (abcam ab150115), and with an anti-MUCIN1 antibody conjugated with Alexa Fluor 546 (Santa Cruz Biotechnologies VU4H5), with an anti-MUC21 antibody (Atlas Antibodies HPA052028) and a secondary antibody conjugated with Alexa Fluor 488 (abcam ab150077). gFBs were stained with an anti-MMP13 antibody (Protein Tech cat.no. 18165-1-AP) and a secondary antibody conjugated with Alexa Fluor 488 (abcam ab150077) and with an anti-VAMP3 antibody and a secondary antibody conjugated with Alexa Fluor 647 (abcam ab150115). Co-localization was observed via fluorescent microscopy. Cell nuclei were stained with DAPI (blue).

Lipid raft isolation

For the isolation of lipid rafts, 2.5×10^6 parental gECs (OKG4) and gEC-VAMP8(-/-) cells were seeded in 10cm petri dishes. At 90% confluence, cells were infected with *E. gingivalis* for 2hrs (MOI=0.0004). Subsequently, the cells were washed 3x with PBS and harvested in 2ml MES lysis buffer and subsequently lysed 25x with the Dounce Homogenizer. 2ml supernatant was then taken up in 2mL 80% sucrose solution (final concentration of 40%). The sucrose solutions were then pipetted into the centrifuge tubes of the corresponding ultracentrifuge. First, 4 mL of the 40% solution was pipetted with the sample contained, then 4 mL of 30 % sucrose solution and finally 4 mL of 5 % sucrose solution (**Appendix Figure 4**). The samples were centrifuged for 18 hrs at 240,000 *g* and 4 °C in an ultracentrifuge using an SW 40 Ti swinging bucket rotor. 1-ml fractions were subsequently collected.

After centrifugation, the centrifugation tubes were carefully removed from the rotor and fractions 3-6, each consisting of 1 mL, were pipetted into 1.5 mL reaction tubes, taking care not to mix the fractions. The fractions were used as a sample for a Western blot to detect the proteins in the lipid rafts.

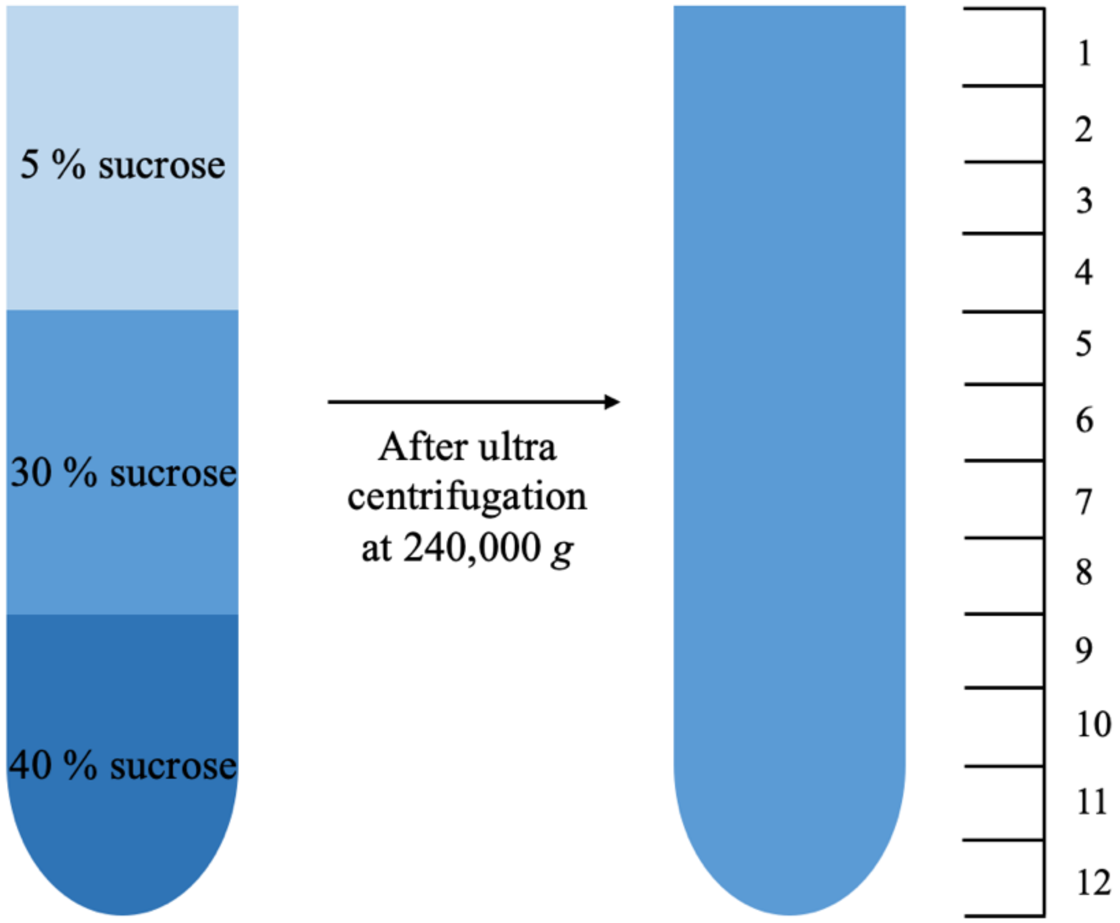

Appendix Figure 4. Ultracentrifuge tubes after loading with the respective sucrose solutions (40 %, 30 % and 5 %).
4 mL per solution were pipetted. The same tube is shown on the right after ultracentrifugation with a schematic representation of the resulting fractions 1-12.

VAMP8 protein content was determined by bicinchoninic acid (BCA, Thermo Fisher) assay according to the manufacturer's instructions. 20 µL of the fractions were mixed with 5 µL of 5x loading buffer and heated at 95°C for 5'. The samples were separated on an SDS gel (30', 200V). The proteins were transferred to a 0.2-µm-pore-size nitrocellulose, blotted (100V, 15'), blocked with PBST–3% BSA, washed with PBST, and incubated with primary VAMP8 antibody (sc-166820, Santa Cruz Biotechnologies) in PBST–3% BSA overnight at 4°C. Aftewards, the membrane was washed 3x with TBS-T and incubated for 1.5h at room temperature and gently swirled in the antibody mixture. The membrane was then washed again with TBS-T and after adding 1ml each of ECL solutions A and B onto the membrane, chemoluminiscence was detected (ChemoStar Touch ECL, INTAS, Germany).

Collagenase-Assay

Parental gFBs and gFB-VAMP3(-/-) cells were cultured to a confluence of 90-95%, infected with *E. gingivalis* or *P. gingivalis* for 2hrs with MOI=0.0004 and MOI=5, respectively and washed 3x. Collagenase activity was quantified using the EnzChek™ Gelatinase/Collagenase Assay Kit (ThermoFisher, E12055) on a microplate spectrophotometer (Multiskan GO, Thermo Fisher) according to the manufacturer's protocol.

Cytotoxicity Assays

Parental gECs and gFB cells and gEC-VAMP8(-/-) and gFB-VAMP3(-/-) cells were cultured to 90-95% confluence in a 96-well plate. Cells were infected with washed *E. gingivalis* or *P. gingivalis* for 2 hrs with MOI=0.0004 and MOI=5, respectively. For control experiments, cells were mock-infected with the supernatant of the washed amoebae or heat-inactivated *P. gingivalis*. 2-hours after infection, dead cells that lost their membrane integrity were quantified by adding the luminogenic peptide Alanyl-Alanyl-Phenylalanyl-Aminoluciferin (AAF-GloTM; CytoTox-Glo™ CytotoxicityAssay, Promega) and luminescence was measured using the microplate luminometer GloMax Explorer (Promega, USA) according to the manufacturer’s instructions.
